# Supplementary material for: The effects of pulmonary rehabilitation on inflammatory biomarkers in patients with chronic obstructive pulmonary disease: Protocol for a systematic review and meta-analysis
Source: PLoS One. 2023 Jun 27;18(6):e0287549. doi: 10.1371/journal.pone.0287549 (PMC10298755; doi:10.1371/journal.pone.0287549)
Supplement: S2 File — (DOCX) [file pone.0287549.s002.docx]

Systematic Review of Impact of Pulmonary Rehabilitation on Systemic Inflammatory Biomarkers in People with COPD: Search Strategies

Table 1: CINAHL

| **Database 1: EBSCOhost Cumulative Index to Nursing and Allied Health Literature Database (CINAHL)** | |
| --- | --- |
| *Concept* | *Search Terms* |
| Chronic obstructive pulmonary disease | 1. (MH “Lung Diseases, Obstructive+”)  2. (MH “Bronchitis+”)  3. (MH “Bronchitis, Chronic”)  4. (MH “Emphysema+”)  5. (MH “Pulmonary Disease Chronic Obstructive+”)  6. “chronic obstructive pulmonary disease*”  7. “copd”  8. “bronchitis”  9. “chronic lung disease*”  10. “emphysema”  11. S1 OR S2 OR S3 OR S4 OR S5 OR S6 OR S7 OR S8  OR S9 OR S10 |
| Pulmonary rehabilitation | 12. (MH “Rehabilitation Pulmonary+”)  13. (MH “Therapeutic Exercise+”)  14. (MH “Exercise+”)  15. “pulmonary rehab*”  16. “exercise*”  17. “respiratory rehab*”  18. “rehab*”  19. S12 OR S13 OR S14 OR S15 OR S16 OR S17 OR S18 |
| Inflammation | 20. (MH “Inflammation”)  21. (MH “Biological Markers”)  22. (MH “Cytokines+”)  23. (MH “Lymphocytes+”)  24. (MH “Leukocytes+”)  25. (MH “Monocytes”)  26. (MH “Eosinophils”)  27. (MH “Neutrophils”)  28. (MH “C-Reactive Protein”)  29. (MH “Interleukins+”)  30. (MH “Proteins+”)  31. (MH “Albumins+”)  32. (MH “Ferritin”)  33. (MH “Iron”)  34. (MH “Blood Platelets”)  35. (MH “Blood Sedimentation”)  36. (MH “Iron Metabolism Disorders+”)  37. (MH “Fibrinogen+”)  38. “inflamma*”  39. “inflammatory mediator*”  40. “systemic inflamma*”  41. “biological marker*”  42. “biomarker*”  43. “cytokine*”  44. “monocyte*”  45. “eosinophil*”  46. “neutrophil*”  47. “c-reactive protein*”  48. “lymphocyte*”  49. “platelet*”  50. “chemokine*”  51. “interleukin*”  52. “protein*”  53. “albumin”  54. “ferritin”  55. “iron*”  56. “iron metabolism”  57. “fibrinogen*”  58. “erythrocyte sedimentation rate”  59. “enzyme-linked immunosorbent assay”  60. “alpha-1 antitrypsin”  61. S20 OR S21 OR S22 OR S23 OR S24 OR S25 OR S26  OR S27 OR S28 OR S29 OR S30 OR S31 OR S32 OR  S33 OR S34 OR S35 OR S36 OR S37 OR S38 OR S39  OR S40 OR S41 OR S42 OR S43 OR S44 OR S45 OR  S46 OR S47 OR S48 OR S49 OR S50 OR S51 OR S52  OR S53 OR S54 OR S55 OR S56 OR S57 OR S58 OR  S59 OR S60 |
| Combined Concepts | 62. S11 AND S19 AND S61 |

Table 2: AMED

| **Database 2: OVID Allied and Complementary Medicine (AMED)** | |
| --- | --- |
| *Concept* | *Search Terms* |
| Chronic obstructive pulmonary disease | 1. exp Pulmonary Disease Chronic Obstructive/  2. exp Pulmonary emphysema/  3. exp Lung diseases obstructive/  4. exp Emphysema/  5. exp Bronchitis/  6. chronic obstructive pulmonary disease.mp.  7. copd.mp.  8. bronchitis.mp.  9. chronic lung disease*.mp.  10. emphysema.mp.  11. S1 OR S2 OR S3 OR S4 OR S5 OR S6 OR S7 OR S8  OR S9 OR S10 |
| Pulmonary rehabilitation | 12. exp Rehabilitation/  13. exp Exercise therapy/  14. exp Exercise/  15. pulmonary rehab*.mp.  16. exercise*.mp.  17. respiratory rehab*.mp.  18. rehab*.mp.  19. S12 OR S13 OR S14 OR S15 OR S16 OR S17 OR S18 |
| Inflammation | 20. Inflammation/  21. biological markers/  22. exp Cytokines/  23. exp Interleukins/  24. proteins/  25. exp Leukocytes/  26. exp Lymphocytes/  27. exp Neutrophils/  28. exp Blood platelets/  29. exp Iron/  30. inflamm*.mp.  31. inflammatory mediator*.mp.  32. biomarker*.mp.  33. biological marker*.mp.  34. cytokine*.mp.  35. chemokine*.mp.  36. interleukin*.mp.  37. protein*.mp.  38. albumin*.mp.  39. ferritin*.mp.  40. alpha-1 antitrypsin.mp.  41. lymphocyte*.mp.  42. monocyte*.mp.  43. eosinophil*.mp.  44. neutrophil*.mp.  45. leukocyte*.mp.  46. platelet*.mp.  47. c-reactive protein*.mp.  48. erythrocyte sedimentation rate*.mp.  49. iron*.mp.  50. iron metabolism*.mp.  51. fibrinogen*.mp.  52. enzyme-linked immunosorbent assay.mp.  53. S20 OR S21 OR S22 OR S23 OR S24 OR S25 OR S26  OR S27 OR S28 OR S29 OR S30 OR S31 OR S32 OR  S33 OR S34 OR S35 OR S36 OR S37 OR S38 OR S39  OR S40 OR S41 OR S42 OR S43 OR S44 OR S45 OR  S46 OR S47 OR S48 OR S49 OR S50 OR S51 OR S52 |
| Combined Concepts | 54. S11 AND S19 AND S53 |

Table 3: EMBASE

| **Database 3: OVID Excerpta Medica Database (EMBASE)** | |
| --- | --- |
| *Concept* | *Search Terms* |
| Chronic obstructive pulmonary disease | 1. exp lung disease/  2. exp chronic lung disease/  3. exp lung emphysema/  4. exp obstructive lung disease/  5. exp chronic obstructive lung disease/  6. exp bronchitis/  7. exp chronic bronchitis/  8. exp emphysema/  9. exp chronic respiratory tract disease/  10. chronic lung disease*.mp.  11. emphysema.mp.  12. bronchitis.mp.  13. copd.mp.  14. chronic respiratory disease*.mp.  15. chronic obstructive pulmonary disease.mp.  16. S1 OR S2 OR S3 OR S4 OR S5 OR S6 OR S7 OR S8  OR S9 OR S10 OR S11 OR S12 OR S13 OR S14 OR  S15 |
| Pulmonary rehabilitation | 17. exp pulmonary rehabilitation/  18. exp rehabilitation/  19. exp exercise/  20. exp kinesiotherapy/  21. therapeutic exercise*.mp.  22. pulmonary rehab*.mp.  23. exercise*.mp.  24. respiratory rehab*.mp.  25. rehab*  26. S17 OR S18 OR S19 OR S20 OR S21 OR S22 OR S23  OR S24 OR S25 |
| Inflammation | 27. inflammation/  28. biological marker/  29. exp cytokine/  30. exp interleukin derivative/  31. protein/  32. albumin/  33. ferritin/  34. alpha-1 antitrypsin/  35. exp leukocyte/  36. exp lymphocyte/  37. exp monocyte/  38. exp eosinophil/  39. exp neutrophil/  40. exp thrombocyte/  41. exp C reactive protein/  42. exp erythrocyte sedimentation rate/  43. exp iron/  44. exp iron metabolism disorder/  45. exp fibrinogen/  46. exp enzyme-linked immunosorbent assay/  47. inflamma*.mp.  48. systemic inflamma*.mp.  49. inflammatory mediator*.mp.  50. biomarker*.mp.  51. biological marker*.mp.  52. cytokine*.mp.  53. chemokine*.mp.  54. interleukin*.mp.  55. protein*.mp.  56. albumin*.mp.  57. ferritin*.mp.  58. alpha-1 antitrypsin*.mp.  59. leukocyte*.mp.  60. lymphocyte*.mp.  61. monocyte*.mp.  62. eosinophil*.mp.  63. neutrophil*.mp.  64. platelet*.mp.  65. c reactive protein*.mp.  66. erythrocyte sedimentation rate*.mp.  67. iron*.mp.  68. iron metabolism*.mp.  69. fibrinogen*.mp.  70. enzyme-linked immunosorbent assay.mp.  71. S27 OR S28 OR S29 OR S30 OR S31 OR S32 OR S33  OR S34 OR S35 OR S36 OR S37 OR S38 OR S39 OR  S40 OR S41 OR S42 OR S43 OR S44 OR S45 OR S46  OR S47 OR S48 OR S49 OR S50 OR S51 OR S52 OR  S53 OR S54 OR S55 OR S56 OR S57 OR S58 OR S59  OR S60 OR S61 OR S62 OR S63 OR S64 OR S65 OR  S66 OR S67 OR S68 OR S69 OR S70 |
| Combined Concepts | 72. S16 AND S26 AND S71 |

Table 4: OVID Medline

| **Database 4: OVID Medline** | |
| --- | --- |
| *Concept* | *Search Terms* |
| Chronic obstructive pulmonary disease | 1. exp Pulmonary Disease, Chronic Obstructive/  2. exp Emphysema/  3. exp Bronchitis, Chronic/  4. exp Bronchitis/  5. exp Lung Diseases, Obstructive/  6. exp Lung Diseases/  7. chronic obstructive pulmonary disease*.mp.  8. copd.mp.  9. bronchitis.mp.  10. emphysema.mp.  11. chronic lung disease*.mp.  12. S1 OR S2 OR S3 OR S4 OR S5 OR S6 OR S7 OR S8  OR S9 OR S10 OR S11 |
| Pulmonary rehabilitation | 13. exp Rehabilitation/  14. exp Exercise/  15. exp Exercise Therapy/  16. pulmonary rehab*.mp.  17. “exercise*”  18. respiratory rehab*.mp.  19. therapeutic exercise*.mp.  20. rehab*.mp.  21. S13 OR S14 OR S15 OR S16 OR S17 OR S18 OR S19  OR S20 |
| Inflammation | 22. Inflammation/  23. Biomarkers/  24. exp Cytokines/  25. exp Interleukins/  26. Proteins/ albumin  27. Ferritins/  28. alpha 1-Antitrypsin/  29. exp Leukocytes/  30. exp Lymphocytes/  31. exp Monocytes/  32. exp Eosinophils/  33. exp Neutrophils/  34. exp Blood Platelets/  35. exp C-Reactive Protein/  36. exp Blood Sedimentation/  37. exp Iron/  38. exp Iron Metabolism Disorders/  39. exp Fibrinogen/  40. exp Enzyme-Linked Immunosorbent Assay/  41. inflamma*.mp.  42. systemic inflamma*.mp.  43. inflammatory mediator*.mp.  44. biomarker*.mp.  45. biological marker*.mp.  46. cytokine*.mp.  47. chemokine*.mp.  48. interleukin*.mp.  49. protein*.mp.  50. albumin*.mp.  51. ferritin*.mp.  52. alpha 1-antitrypsin*.mp.  53. leukocyte*.mp.  54. lymphocyte*.mp.  55. monocyte*.mp.  56. eosinophil*.mp.  57. neutrophil*.mp.  58. platelet*.mp.  59. c-reactive protein*.mp.  60. erythrocyte sedimentation rate.mp.  61. iron*.mp.  62. iron metabolism*.mp.  63. fibrinogen*.mp.  64. enzyme-linked immunosorbent assay.mp.  65. S22 OR S23 OR S24 OR S25 OR S26 OR S27 OR S28  OR S29 OR S30 OR S31 OR S32 OR S33 OR S34 OR  S35 OR S36 OR S37 OR S38 OR S39 OR S40 OR S41  OR S42 OR S43 OR S44 OR S45 OR S46 OR S47 OR  S48 OR S49 OR S50 OR S51 OR S52 OR S53 OR S54  OR S55 OR S56 OR S57 OR S58 OR S59 OR S60 OR  S61 OR S62 OR S63 OR S64 |
| Combined Concepts | 66. S12 AND S21 AND S65 |

Table 5: MEDLINE (Pubmed)

| **Database 1: MEDLINE (Pubmed)** | |
| --- | --- |
| *Concept* | *Search Terms* |
| Chronic obstructive pulmonary disease | 1. exp Pulmonary Disease, Chronic Obstructive/  2. exp Emphysema/  3. exp Bronchitis, Chronic/  4. exp Bronchitis/  5. exp Lung Diseases, Obstructive/  6. exp Lung Diseases/  7. chronic obstructive pulmonary disease*.mp.  8. copd.mp.  9. bronchitis.mp.  10. emphysema.mp.  11. chronic lung disease*.mp.  12. S1 OR S2 OR S3 OR S4 OR S5 OR S6 OR S7 OR S8  OR S9 OR S10 OR S11 |
| Pulmonary rehabilitation | 13. exp Rehabilitation/  14. exp Exercise/  15. exp Exercise Therapy/  16. pulmonary rehab*.mp.  17. “exercise*”  18. respiratory rehab*.mp.  19. therapeutic exercise*.mp.  20. rehab*.mp.  21. S13 OR S14 OR S15 OR S16 OR S17 OR S18 OR S19  OR S20 |
| Inflammation | 22. Inflammation/  23. Biomarkers/  24. exp Cytokines/  25. exp Interleukins/  26. Proteins/ albumin  27. Ferritins/  28. alpha 1-Antitrypsin/  29. exp Leukocytes/  30. exp Lymphocytes/  31. exp Monocytes/  32. exp Eosinophils/  33. exp Neutrophils/  34. exp Blood Platelets/  35. exp C-Reactive Protein/  36. exp Blood Sedimentation/  37. exp Iron/  38. exp Iron Metabolism Disorders/  39. exp Fibrinogen/  40. exp Enzyme-Linked Immunosorbent Assay/  41. inflamma*.mp.  42. systemic inflamma*.mp.  43. inflammatory mediator*.mp.  44. biomarker*.mp.  45. biological marker*.mp.  46. cytokine*.mp.  47. chemokine*.mp.  48. interleukin*.mp.  49. protein*.mp.  50. albumin*.mp.  51. ferritin*.mp.  52. alpha 1-antitrypsin*.mp.  53. leukocyte*.mp.  54. lymphocyte*.mp.  55. monocyte*.mp.  56. eosinophil*.mp.  57. neutrophil*.mp.  58. platelet*.mp.  59. c-reactive protein*.mp.  60. erythrocyte sedimentation rate.mp.  61. iron*.mp.  62. iron metabolism*.mp.  63. fibrinogen*.mp.  64. enzyme-linked immunosorbent assay.mp.  65. S22 OR S23 OR S24 OR S25 OR S26 OR S27 OR S28  OR S29 OR S30 OR S31 OR S32 OR S33 OR S34 OR  S35 OR S36 OR S37 OR S38 OR S39 OR S40 OR S41  OR S42 OR S43 OR S44 OR S45 OR S46 OR S47 OR  S48 OR S49 OR S50 OR S51 OR S52 OR S53 OR S54  OR S55 OR S56 OR S57 OR S58 OR S59 OR S60 OR  S61 OR S62 OR S63 OR S64 |
| Combined Concepts | 66. S12 AND S21 AND S65 |
